# Grey Squirrel Density and Damage in Relation to the Presence of Predators

**DOI:** 10.3390/ani16213394

**Published:** 2026-10-27

**Authors:** Cally Ham, Eleanor R. Scopes, Nicola Rae, Josh Weaver, Robin Gill

**Affiliations:** 1 Forest Research, Alice Holt Lodge, Farnham GU10 4LH, UK; josh.weaver@forestresearch.gov.uk (J.W.);; 2 Forest Research, Forestry Commission National Office, Bristol BS16 1EJ, UK; ellie.scopes@forestresearch.gov.uk; 3 Environment and Sustainability Institute, University of Exeter, Penryn TR10 9FE, UK; 4 Forest Research, Northern Research Station, Edinburgh EH25 9SY, UK; nicola.rae@forestresearch.gov.uk

**Keywords:** grey squirrel, pine marten, goshawk, predator–prey, *Sciurus carolinensis*, bark stripping, woodland management

## Abstract

The grey squirrel (*Sciurus carolinensis*) is an invasive species in Great Britain that causes substantial economic and ecological damage through stripping bark from broadleaf trees. The relationship between grey squirrel abundance and tree damage remains poorly understood, as does the potential role of recovering native predators in mitigating these impacts. We conducted an observational survey of 22 woodland sites across England and Wales with differing occurrences of pine martens (*Martes martes*) and northern goshawks (*Accipiter gentilis*), estimating grey squirrel density and quantifying bark-stripping damage. Grey squirrel density varied across sites. No significant relationship was detected between squirrel density and observed tree damage when analyses were restricted to sites where squirrels were present. However, when sites with no detected squirrels were included, squirrel density was positively associated with tree damage. Predator occurrence was associated with significantly lower grey squirrel densities. In contrast, predator presence was not significantly associated with the proportion of trees exhibiting bark-stripping damage. These findings suggest that predators may contribute to grey squirrel population suppression but that bark stripping is influenced by factors beyond contemporary squirrel abundance. Forest management practices that facilitate predator presence should be encouraged to support reduction in grey squirrel density and therefore improve future tree health. Further research should focus on fresh damage assessments and predator-mediated behavioural responses to better understand the mechanisms linking predator recovery, squirrel abundance and tree damage.

## Simple Summary

Grey squirrels are a non-native species in Great Britain that can damage broadleaf trees by stripping away bark. This reduces timber quality, harms tree health and creates significant costs for woodland owners. Although controlling grey squirrel populations is a common management practice, it is unclear whether lower squirrel numbers lead directly to less tree damage. We surveyed 22 woodlands across England and Wales, with different occurrences of two natural predators, pine martens and goshawks, to estimate grey squirrel numbers and measure bark-stripping damage. We found that woodlands with predators generally had fewer grey squirrels than sites without predators. However, lower squirrel numbers were not associated with clear reductions in visible tree damage. This may be because bark damage remains visible for many years, meaning that current squirrel numbers do not necessarily reflect the populations that originally caused the damage. Our findings suggest that natural predators may help reduce grey squirrel populations, but any benefits for tree health may take time to become apparent. Improving our understanding of how predators influence both grey squirrel numbers and behaviour could support more effective and sustainable woodland management and help reduce the impacts of this invasive species on British forests.

## 1. Introduction

The eastern grey squirrel (*Sciurus carolinensis*) was introduced to Great Britain on multiple occasions between 1876 and 1929 and subsequently spread rapidly throughout the country [1]. The most recent estimates suggest that Britain supports approximately 2.7 million grey squirrels, of which around 1.94 million occur in England [2]. Since their introduction, grey squirrels have become one of the most widespread and economically significant invasive mammals in Britain, with substantial impacts on both woodland ecosystems and forestry production [3].

The most well-documented ecological consequence of grey squirrel expansion has been the decline of the native red squirrel (*Sciurus vulgaris*) throughout most of England, Wales and southern Scotland. This decline is driven by a combination of exploitative competition for resources [4] and the transmission of squirrelpox virus, which is typically asymptomatic in grey squirrels but frequently fatal to red squirrels [5]. Beyond impacts on red squirrels, grey squirrels may also influence woodland composition and management. Persistent bark-stripping damage can reduce the viability of broadleaved forestry, potentially altering tree species selection, reducing woodland productivity, and discouraging long-term woodland management [6]. The broader ecological consequences of these changes remain poorly understood.

Grey squirrels cause considerable economic damage through bark stripping, particularly on sycamore (*Acer pseudoplatanus*), beech (*Fagus sylvatica*) and birch (*Betula* spp.) trees between 10 and 40 years of age [7,8,9]. Removal of bark can expose trees to pathogens, cause structural deformities and branch failure, and ultimately result in tree mortality [10,11]. Consequently, bark stripping reduces both the quantity and quality of timber production, compromises carbon sequestration potential, and necessitates costly management interventions. Across England and Wales, the annual economic cost of grey squirrel impacts has been estimated at approximately £37 million [12].

Despite decades of research, the drivers of bark-stripping behaviour remain unclear. Nutritional hypotheses propose that squirrels remove bark to access phloem sugars or obtain minerals such as calcium [13,14], whereas behavioural explanations suggest that bark stripping may emerge in response to negative social interactions or elevated population density, especially amongst juveniles [9,13]. The relationship between squirrel abundance and observed levels of tree damage remains uncertain. The lack of mechanistic understanding constrains management as current control efforts are largely directed at reducing population size rather than targeting the behavioural drivers of damage.

Across Britain, grey squirrel management currently relies on lethal control through trapping and shooting [15]. Population reduction is generally assumed to lessen bark-stripping damage and improve woodland condition, yet empirical evidence linking squirrel density with levels of tree damage remains limited. Moreover, changes in population size may alter environmental impacts through both direct numerical effects and indirect behavioural responses [16]. Harvesting and predation can modify space use, foraging activity and habitat selection, thereby influencing the extent to which animals interact with vulnerable resources. For example, hunting pressure has been shown to alter herbivory patterns in black-tailed deer (*Odocoileus hemionus sitkensis*) through behaviourally mediated responses to predation risk [17].

Such behavioural responses are frequently described within the framework of the “landscape of fear”, whereby prey adjust their spatial and temporal behaviour in response to perceived predation risk [18]. These responses can include increased vigilance, shifts in habitat use, and reduced foraging activity in areas perceived as dangerous [19,20,21]. Importantly, predators can therefore influence ecosystems not only through direct reductions in prey abundance but also through changes in prey behaviour. If bark stripping is partly driven by behavioural processes, predator-induced changes in grey squirrel behaviour could potentially reduce tree damage even in the absence of substantial reductions in squirrel density.

This possibility is particularly relevant given the recent reintroduction of native predators in Britain. Pine martens (*Martes martes*) have been reintroduced to parts of England and Wales [22,23,24] and are widely anticipated to contribute to grey squirrel suppression, as documented previously in Ireland and Scotland [25,26]. Evidence from Wales suggests that pine marten presence may alter grey squirrel ranging behaviour, consistent with a landscape-of-fear effect [27]. However, experimental studies have reported weaker antipredator responses by grey squirrels to pine marten scent compared with native red squirrels, suggesting that behavioural responses may be limited or context-dependent [28]. Consequently, the extent to which pine martens influence grey squirrel behaviour and associated woodland impacts remains uncertain.

Northern goshawks (*Accipiter gentilis*) represent another increasingly important predator of grey squirrels in British woodlands. Following population recovery, goshawks have become more widespread across England and Wales [29], and dietary studies indicate that grey squirrels can constitute a substantial component of breeding-season prey [30,31]. As goshawk breeding coincides with the emergence of juvenile squirrels from dreys, predation pressure may be particularly influential during this period. However, despite evidence of predation, little is known about whether goshawks affect grey squirrel abundance, behaviour, or subsequent levels of bark stripping.

Given the improbability of national grey squirrel eradication and the high costs associated with sustained lethal control, identifying mechanisms that reduce the impacts of grey squirrels on woodlands is of considerable ecological and practical importance. In particular, understanding whether predators influence bark-stripping damage through direct population suppression or behaviourally mediated effects could inform more efficient and ecologically sustainable woodland management strategies.

Despite extensive research on grey squirrel population control and bark-stripping damage, few studies have simultaneously evaluated contemporary squirrel density, predator occurrence and tree damage within the same woodland sites. In particular, it remains unclear whether predator-associated reductions in squirrel density are reflected in measurable differences in bark-stripping damage.

Here, we investigate the relationships among grey squirrel abundance, predator occurrence and tree damage in British woodlands. Specifically, we aim to determine:

(1) If there is a relationship between grey squirrel density and observable tree damage;
(2) If there is an effect of grey squirrel predators (pine marten and goshawk) on the population density of grey squirrels;
(3) If there is an effect of grey squirrel predators (pine marten and goshawk) on the amount of observable tree damage.

We predicted that (H1) higher grey squirrel density would be associated with greater bark-stripping damage; (H2) sites with predator occurrence would support lower grey squirrel densities than predator-free sites; and (H3) predator occurrence would be associated with lower levels of fresh and/or total bark-stripping damage.

As behavioural variables are not directly measured in this study, the landscape-of-fear mechanism is evaluated only indirectly and should therefore be considered a proposed explanatory mechanism rather than a tested causal pathway.

We anticipate that the results presented here will provide insight into designing effective grey squirrel management strategies, ultimately contributing to improved tree and woodland health and productivity.

## 2. Materials and Methods

### 2.1. Site Selection and Information

All study sites were situated within publicly owned forests managed by either Forestry England (15 sites) or Natural Resources Wales (9 sites), located at least 1 km apart, and were recorded as having grey squirrels present. Study sites were selected from the national forest databases for England and Wales using the following criteria: areas > 5 ha with >50% of the trees recorded as oak (*Quercus robur* or *Q. petraea*), beech (*Fagus sylvatica*) or sycamore (*Acer pseudoplatanus*), and aged between 10 and 40 years (Table S1). These tree species were selected as they are most vulnerable to grey squirrel bark stripping [14] and would be most sensitive to changes due to the presence of predators. The chosen sites are all considered “commercial forestry” and were planted with the predominant aim of producing timber. Such woodlands are known to be used by grey squirrels, pine marten [32,33] and goshawk [30,34] but represent a lower tree species diversity than surrounding areas of native broadleaf. Reliable information on current and past grey squirrel control were not available. It is possible that prior control efforts could impact the results; however, it is unlikely that this would cause a systematic bias, as grey squirrel control is local.

Goshawk and pine marten presence data were obtained from Forestry England and Natural Resources Wales in the form of recorded sightings and location of known goshawk nests. Goshawk data reflected either sightings or nests recorded in the two years prior to the field surveys; whilst not all nests are likely to have been occupied, nest presence likely indicates recent goshawk activity within the area. Pine marten presence data from Forestry England and Natural Resources Wales reflected verified sightings (for example, through confirmed camera-trap sightings) from the year prior to field surveys. Further information on pine marten presence was obtained from The Vincent Wildlife Trust Marten Map Explorer with location data updated annually [35].

### 2.2. Grey Squirrel Density Estimate

An existing methodology, which uses squirrel images as a proxy estimate of squirrel density, was used [36]. This method assumes that the attractiveness of the bait remains consistent across sites, and that the bait increases the consistency of detections, such that a direct link between detection rates and abundance can be drawn [36]. Camera traps (Browning Patriot Trail Camera, Browning Trail Cameras, Birmingham, AL, USA) were deployed one per hectare (in accordance with the published methodology) within each of the chosen sites (Figure S1). Using ArcGIS (ArcMap 10.6.1), a 1 ha grid of random origin was laid over the site and the camera location was taken as the central point of each square (Figure S1). Camera traps were deployed between June and August of 2023 and 2024, when food availability is relatively low and so the bait is likely to be attractive to grey squirrels [36]. For sites larger than 10 ha, a subsection was used. Squirrel density was calculated at a site-level based on the number of grey squirrel images per camera per day.

Cameras were attached to the nearest suitable tree from the predetermined location, positioned at 1 m in height and angled downward to view the bait pile. The bait pile, consisting of 750 g of peanuts and 750 g of whole maize, was positioned 1–2 m in front of each camera.

Camera traps were programmed with high sensitivity to record three images per detection and with a three-second delay between triggers. Cameras automatically used infrared when on-board sensors detected low light levels.

Images collected between the time of camera deployment and the following midnight were discounted. Images gathered from the first midnight for five consecutive days were used for analysis [36]. Image counting was discontinued if either the position of the camera moved so that the bait pile was out of view or if the bait pile was completely consumed (93/204 camera deployments). Following an unsuccessful trial of AI-assisted image processing, images were manually tagged to record instances of grey squirrel presence. From the tagged images, “squirrel bouts” were calculated, defined as the period of time from initial grey squirrel identification until five minutes following the final grey squirrel detection within a sequence. Following the methods of Beatham et al. (2023) [36], the number of grey squirrel bouts detected at a site per camera per day were calculated and entered into Equation (1) to convert this number into grey squirrel density per hectare for each site. The constant values within the equation were derived from previously published methodology based upon the relationship between camera-trap records and squirrels removed during culling operations. The published method does not supply information to enable calculation of confidence intervals and so here we present only the estimated density value.

(1) $$0.8203\;+\;0.4446\;\times \;bouts\;\left(per\;camera,\;per\;day\right)$$

Equation (1) provided by Beatham et al. (2023) [36] for converting the number of grey squirrel images detected per camera per day into a density estimate.

### 2.3. Tree Damage Assessment

The Nearest Neighbour Method [37] was used to assess tree damage. Surveys were conducted once deciduous trees had dropped their leaves, between November 2023 and February 2024 and November 2024 and February 2025. Following the Nearest Neighbour Method, 20 clusters, each of five trees were assessed per site (giving a total of 100 trees assessed per site). The distance between clusters was determined using Equation (2).

(2) $$Distance\;(m)=\frac{\sqrt{Area\;\left(ha\right)\times 10,000}}{20}$$

Equation (2), distance between surveyed tree clusters, based upon the published Nearest Neighbour Method.

For each site, the first cluster location was at the southwest point with the next cluster located at the derived distance from the first cluster in a northern direction. Subsequent clusters were located the derived distance from the previous cluster; once the edge of the site was reached, a second row of survey clusters was mapped, with the same distance between clusters maintained [37].

For each tree, all visible bark-stripping damage was assessed, with each discrete damage patch recorded according to its age, location, and size, thereby providing a count of damage patches within each category (Table 1). Damage age was recorded because bark-stripping scars can remain visible for many years, undergoing progressive changes in colour and texture over time [38,39]. Categorising damage by age therefore enables differentiation between recent and historic impacts and provides insight into temporal patterns of grey squirrel activity. Damage assessments were conducted by two fieldworkers and age classification confirmed by both assessors to promote inter-observer reliability.

Trees were also assessed for structural impacts attributable to grey squirrel damage: (1) snapped crown, where the upper section of the main stem was absent due to stem failure associated with bark stripping; (2) distorted main stem, where the main stem exhibited bending, forking, or splitting resulting from damage-related growth deformities; and (3) ringbarking (girdling), where bark had been removed around the entire circumference of the main stem. Only trees with a diameter at breast height (DBH) greater than 5 cm were included in the assessment, as smaller trees were considered unlikely to exhibit damage attributable to grey squirrel bark stripping.

### 2.4. Data Analysis

All data analysis was conducted in R version 4.5.2 [40].

Predator occurrence was categorised in two ways, firstly as a variable with four levels (neither goshawk nor pine marten present, both goshawk and pine marten present, only goshawk present and only pine marten present). Due to unbalanced sample sizes, a second dataset was generated using a binary variable of “predators present” (when goshawk and/or pine marten were present) and “predators absent” (when goshawk and pine marten were both absent) to facilitate further analysis.

Two datasets were used throughout the analyses. Firstly, a dataset including all sites surveyed. Secondly, a dataset including only those sites where grey squirrels were observed; this was used to assess whether relationships observed across all sites were maintained among only squirrel-occupied sites.

#### 2.4.1. Survey Year and Tree Species

Differences in grey squirrel density between survey years and between predominant tree-species classes were assessed using non-parametric Wilcoxon rank-sum tests because assumptions of normality were not consistently met due to uneven class distribution across tree species and unequal distribution of predator presence across the two survey years.

#### 2.4.2. Predator Presence and Grey Squirrel Density

Relationships between predator presence and grey squirrel density were initially explored using linear mixed-effects models fitted with the lmerTest package [41], with predator category included as a fixed effect and survey year included as a random intercept. Because only two survey years were available, random-effect variance was poorly estimated and confidence intervals included zero. Consequently, survey year was subsequently treated as a fixed effect within linear models fitted using the stats package [40]. Habitat was also not included in these models because tree species classes were highly unbalanced (oak = 15 sites, beech = 6 sites, sycamore = 1 site), partially confounded with predator occurrence, preventing reliable estimation of independent habitat effects. Each of the chosen tree species (oak, beech and sycamore) is associated with high levels of squirrel bark stripping in England and Wales [7,8,9]; therefore, excluding these habitat types from the analyses should not influence the results. Model assumptions were assessed using diagnostic plots of residuals against fitted values, normal quantile–quantile plots, Cook’s distance, leverage statistics, Shapiro–Wilk tests of residual normality, and Breusch–Pagan tests of heteroscedasticity using the lmtest [42]. Where severe imbalance among predator categories resulted in influential observations and unstable parameter estimates, predator occurrence was simplified to the binary variable—predators present or predators absent.

#### 2.4.3. Predator Presence and Grey Squirrel Tree Damage

Tree damage was quantified using two response variables: (1) proportion of trees at a site with signs of fresh squirrel damage and (2) proportion of trees at a site with any sign of squirrel damage. Exploratory analyses showed that proportion of trees with fresh damage was highly zero-inflated with most sites not having signs of fresh damage. Therefore, standard Gaussian models were considered inappropriate for this variable and instead a binary variable of “fresh damage present” or “fresh damage absent” was used for each site.

Non-parametric analyses were used to assess the effect of predators on tree damage because damage data are bounded proportions and sample sizes among predator categories small and unbalanced. To assess the differences in tree damage against the predator categorical variable with four levels (neither goshawk nor pine marten present, both goshawk and pine marten present, goshawk only present and pine marten only present) the Kruskal–Wallis rank-sum test [40] was used. To assess differences in tree damage against the binary predator present or predators absent variable, the Wilcoxon rank-sum test [40] was used.

Where the fresh squirrel damage variable was converted to a binary response (present versus absent) at a site level, use of generalised linear models with binomial error structure [40] was explored. However, these analyses exhibited complete or near-complete separation because damage was absent from some predator classes. Consequently, inference from these models was considered unreliable and only descriptive summaries were retained.

#### 2.4.4. Grey Squirrel Density and Tree Damage

To assess whether squirrel abundance was associated with damage levels, relationships between squirrel density and the proportion of damaged trees were analysed using both non-parametric and parametric approaches.

Monotonic associations were evaluated using Spearman rank correlations [40]. Linear relationships were examined using ordinary least-squares regression [40], with squirrel density as the explanatory variable and tree damage as the response variable. Models were fitted both to the squirrel-only subset and to the full dataset to determine whether sites without grey squirrel detections influenced the observed relationships.

For linear models, parameter estimates, standard errors, coefficients of determination (*R*^2^), and 95% confidence intervals are reported. Model assumptions were assessed using the diagnostic procedures described above.

## 3. Results

### 3.1. Field Surveys

A total of 40,631 images from 12 sites in 2023 and 256,148 images from 12 sites in 2024 (Table S1) were collected. Due to site access difficulties, two sites in 2023 were only 3 ha. Unfortunately, it was not possible to conduct damage surveys at two sites due to forestry activities and restricted access, so these sites were removed from the study, leaving 22 sites for the analysis.

### 3.2. Grey Squirrel Densities

The surveyed sites had a range of grey squirrel densities, up to almost seven individuals/hectare (Table 2). At four sites, we did not detect any grey squirrels using the camera traps and so estimated grey squirrel density as zero for those sites.

### 3.3. Survey Year and Tree Species

Squirrel density differed significantly between survey years, Wilcoxon rank-sum test: *W* = 118, *p* < 0.001.

Potential habitat effects were assessed using a Wilcoxon rank-sum test [40] comparing oak- and beech-dominated sites following exclusion of the single sycamore-dominated site. Predominant tree species showed only weak evidence of an association with squirrel density: Wilcoxon rank-sum test: *W* = 70, *p* = 0.056.

### 3.4. Grey Squirrel Density and Tree Damage

When analyses were restricted to sites where grey squirrels were observed (n = 18), there was no evidence that grey squirrel density had a significant influence on the total observed squirrel damage (including fresh and old damage): Spearman’s rank correlation (ρ = 0.41, *p* = 0.089) and linear regression (β = 0.084 ± 0.171 SE, *p* = 0.628, *R*^2^ = 0.015).

However, when all sites were included (including the four sites without grey squirrel observations, n = 22), a stronger association between squirrel density and the total observed squirrel damage emerged: Spearman’s rank correlation (ρ = 0.673, *p* < 0.001) and linear regression indicates that tree damage increased significantly with squirrel density (β = 0.312 ± 0.107 SE, 95% CI = 0.088–0.535, *t* = 2.91, *p* = 0.009) (Figure 1). The linear regression model explained almost 30% of the variation in recorded tree damage: *R*^2^ = 0.298, *F*_1,20_ = 8.47, *p* = 0.009.

Assessing the relationship between grey squirrel density and observed fresh tree damage proved to be unachievable with our datasets due to fresh damage only being observed at six sites and absent from the predator-free sites. Therefore, formal inference from the attempted models was considered inappropriate.

### 3.5. Predator Presence and Grey Squirrel Density

Two sites had no data on goshawk presence and were therefore excluded from the analyses relating to goshawk presence (n = 16).

Predator occurrence was associated with substantial variation in squirrel density. Initial analyses treating predator status as four categories (neither present, both present, goshawk only, pine marten only) were constrained by highly unbalanced sample sizes (neither present = two sites, both present = nine sites, goshawk only = four sites, pine marten only = one site) and violations of model assumptions (non-normal residuals, heteroscedasticity and influential observations). Consequently, predator occurrence was simplified to a binary variable (predators present or absent) for the primary analysis.

A linear model [40], excluding the year variable, showed that sites where predators were present had significantly lower squirrel densities than predator-free sites (β = −1.35 ± 0.33 SE, 95% CI = −2.05 to −0.65, *t* = −4.04, *p* < 0.001) (Figure 1). The model explained 47.6% of the variation in squirrel density (*R*^2^ = 0.476, *F*_1,18_ = 16.36, *p* < 0.001).

Residuals deviated from normality (Shapiro–Wilk test: *W* = 0.735, *p* < 0.001), although there was no evidence of heteroscedasticity in the simplified model (Breusch–Pagan test: BP = 0.38, *p* = 0.537). The binary predator model was therefore considered more robust than the original four-category analysis.

### 3.6. Predator Presence and Grey Squirrel Tree Damage

The proportion of trees recorded with any visible damage at a site was assessed in relation to predator presence (using the variable with four categories), and suggested there was no significant difference among predator categories (Kruskal–Wallis test: χ^2^ = 4.73, df = 3, *p* = 0.193).

Similarly, when the simplified binary predator variable was used, there was no significant difference between predator presence and predator absence sites (Wilcoxon rank-sum test: *W* = 26, *p* = 0.344). The mean proportion of trees with recorded damage did vary between predator present and predator absent sites (predator absent: 0.60 ± 0.04 SD (*n* = 2), predator present: 0.38 ± 0.33 SD (*n* = 18)). The small number of predator absent sites likely reduces the statistical power to detect predator effects on damage.

## 4. Discussion

This study sought to determine whether grey squirrel bark-stripping damage in English and Welsh woodlands is related to grey squirrel density and whether the presence of key predators, pine martens and goshawks, influences either grey squirrel abundance or the extent of tree damage. Our results provide observational evidence that predator occurrence may be associated with substantially lower grey squirrel densities but does not provide any evidence to suggest a direct relationship between predator presence and observable tree damage. Furthermore, the relationship between squirrel density and observed tree damage appears to depend on whether sites with no detectable grey squirrels are included in the analysis, highlighting the complexity of linking contemporary squirrel abundance to observable evidence of tree damage within a purely observational study design.

### 4.1. Grey Squirrel Density and Tree Damage

Grey squirrel density was not significantly associated with the amount of observable tree damage when analyses were restricted to sites where grey squirrels were present. However, when sites with no detected squirrels were included, a significant positive relationship emerged, with squirrel density explaining approximately 30% of the variation in tree damage.

This apparent contradiction likely reflects the cumulative nature of bark-stripping damage. Once bark stripping has occurred, scars may remain visible for many years [32], meaning that current squirrel density may not accurately reflect the population density that existed when damage was initially inflicted. Consequently, sites supporting similar contemporary squirrel densities may exhibit markedly different levels of visible damage depending on historical squirrel abundance, woodland management practices, stand age, and tree species composition. The stronger relationship observed when sites without squirrels observed were included suggests that variation in observed tree damage is likely driven by multiple factors, not only squirrel density. As with other herbivory observations [43], it is likely that landscape-scale factors such as tree species composition in the wider area, habitat connectivity and anthropogenic pressure also influence grey squirrel bark stripping. Further information on current and past squirrel control within the local area would enable greater interpretation of these results.

These findings support previous suggestions that bark stripping is not solely a density-dependent phenomenon [9,13]. If bark stripping is influenced by nutritional requirements, resource availability, population structure, or individual behavioural differences, then similar densities of squirrels could generate substantially different levels of damage. The weak explanatory power of squirrel density within occupied sites suggests that abundance alone may be an inadequate predictor of woodland impacts and that future studies should attempt to incorporate measures of food availability, woodland composition, and squirrel demography.

An important limitation of the present study is the inability to analyse fresh bark-stripping damage. Fresh damage represents the most appropriate measure for examining relationships with contemporary squirrel densities because it more closely reflects current behaviour. However, fresh damage was observed at only six sites and was absent from predator-free sites, preventing robust statistical inference. Longitudinal studies that repeatedly measure fresh damage over several years could provide a more direct test of whether reductions in squirrel abundance lead to corresponding reductions in bark stripping.

Additional site information such as previous tree-thinning activities and tree species composition in the surroundings may also help to explain the variation in damage and density recorded here.

### 4.2. Predator Occurrence and Grey Squirrel Density

The most consistent result from this study was the negative association between predator occurrence and grey squirrel density. Sites where pine martens and/or goshawks were present supported significantly lower squirrel densities, with predator occurrence explaining nearly half of the observed variation in squirrel abundance.

This finding is consistent with previous work demonstrating population-level suppression of grey squirrels in areas occupied by pine martens in Scotland and Ireland [25,26]. These studies [25,26] found a stronger negative correlation between pine marten presence and grey squirrel presence then presented here, as they have larger sample sizes. Although our study could not statistically separate the effects of pine martens from those of goshawks because of limited and highly unbalanced sample sizes, the observed pattern suggests that predation pressure may be an important determinant of grey squirrel abundance across British woodlands.

Several mechanisms may explain the lower squirrel densities observed at predator-present sites. Direct mortality is the most obvious explanation, particularly given evidence that both pine martens and goshawks prey upon grey squirrels [26,30,31]. However, predator presence may also influence population dynamics through behavioural pathways. Increased vigilance, altered habitat use, reduced foraging efficiency, and avoidance of high-risk areas could lower local carrying capacity or increase emigration from predator-occupied woodlands. Such mechanisms are consistent with the landscape-of-fear framework [18] and suggest that predator effects on grey squirrel populations may extend beyond direct consumption. Furthermore, the method used for assessing grey squirrel density involving the deployment of camera traps positioned over a bait-pile on the floor might be less likely to detect grey squirrels exhibiting greater predator avoidance as they are likely to spend less time exposed on the ground when predation risk is greater [44].

Nevertheless, caution is warranted when interpreting these results. Predator occurrence was simplified to a binary variable because of unbalanced sample sizes, and model residuals showed substantial departures from normality. Although the predator effect remained strong despite these limitations, future studies with larger sample sizes and more balanced representation of predator categories would allow a more rigorous examination of species-specific effects and potential interactions between predators. Furthermore, all study sites visited in 2024 had predators present, while the study sites visited in 2023 had a mixture of predator presence and absence. This makes it impossible to rule out the possibility that grey squirrel density is driven solely by annual variation, rather than predator presence.

### 4.3. Predator Occurrence and Tree Damage

Despite the negative association between predators and squirrel density, there was no statistically significant relationship between predator occurrence and the proportion of trees exhibiting visible damage. This result suggests that any predator effects on squirrel abundance do not necessarily translate into immediately detectable reductions in cumulative bark-stripping damage or that predators have not been exerting an influence on the grey squirrels for long enough to produce an observable effect. Elsewhere, pine marten impact on grey squirrel density increases when pine martens are at higher density and/or they have been established for a longer period of time [26,45].

The lack of a significant result may also reflect limited statistical power. Only two sites were classified as predator-absent, compared with eighteen predator-present sites. Although mean damage levels were numerically higher at predator-free sites, the imbalance in sample sizes substantially reduced our ability to detect differences statistically. Therefore, the absence of a significant effect should not be interpreted as evidence that predators have no influence on woodland damage but should highlight areas for future empirical research.

There may be a time lag between predator recovery and impacts on woodland health. Pine martens and goshawk populations are recovering after historical lows in the UK and are unlikely to have reached carrying capacity. As predator densities increase, there may be greater impacts on grey squirrel bark-stripping behaviour.

Another possibility is that predator-mediated reductions in squirrel density (at the levels observed in our study sites) are insufficient, on their own, to reduce bark stripping. If a relatively small number of individuals, for example, juveniles, account for a disproportionate amount of damage [13], then substantial reductions in population size may be required before changes in damage become detectable. Alternatively, bark stripping may be influenced more strongly by behavioural or nutritional drivers than by overall abundance.

### 4.4. Implications for the Landscape-of-Fear Hypothesis

One objective of this study was to assess whether predators might indirectly reduce bark stripping through behavioural changes associated with perceived predation risk. Our findings provide only indirect support for this possibility. Predator occurrence was associated with lower squirrel densities, but there was no corresponding reduction in observed tree damage.

Several interpretations are possible. First, predators may primarily exert direct numerical effects through mortality rather than inducing strong behavioural changes. Second, behavioural responses may occur but not affect bark-stripping behaviour specifically. Third, any behavioural changes could be masked by the long persistence of historical damage. Given the mixed evidence regarding grey squirrel responses to pine marten predation risk [27,28], further work is required to determine whether landscape-of-fear effects meaningfully influence bark stripping.

Future studies could investigate this question more directly by comparing rates of fresh bark stripping, activity budgets, habitat selection, and movement patterns across gradients of predator activity. Such approaches would provide stronger mechanistic evidence than analyses based solely on cumulative measures of damage using an observational design.

### 4.5. Limitations

This study has small sample sizes and imbalanced representation across predator categories and presence, which has limited the statistical analysis. Although the analysis is robust and demonstrates the impact of predators on grey squirrel density and grey squirrel abundance on cumulative damage, further studies with greater sample sizes would strengthen the conclusions. Future studies could also ensure that annual fluctuations in grey squirrel numbers are not influencing the results. Although it is unlikely to cause a systematic bias, this study could not account for the history of grey squirrel control at the sampled sites, and this may have obscured some of the links between predators, density and damage. This study also relied on existing distribution records to identify the predators present at a site, which potentially could mis-assign sites to the different predator categories and impact the results. 

## 5. Conclusions

From a management perspective, these findings suggest that predator restoration may contribute to reducing grey squirrel populations, supporting the growing interest in the ecological role of recovering pine marten [22,23] and goshawk populations. However, the lack of a clear relationship between predator occurrence and observed tree damage indicates that reductions in squirrel abundance alone may not immediately translate into measurable improvements in woodland condition. As predator populations continue to expand and recover, managers could monitor fresh damage to assess whether higher predator densities reduce further impacts of grey squirrels on tree health. Monitoring data on a large scale could help identify what predator density is required to meaningfully prevent bark stripping. Overall, this study demonstrates that predator presence is associated with substantially lower observed grey squirrel densities but provides limited evidence that either squirrel density or predator occurrence directly predicts bark-stripping damage. The results highlight the importance of considering temporal lags, historical damage legacies, and behavioural mechanisms when assessing the impacts of invasive grey squirrels. Future research should focus on quantifying fresh bark-stripping damage and examining behavioural responses to predation risk to better understand how predator recovery may influence both grey squirrel populations and their impacts on British woodlands.

## Figures and Tables

**Figure 1:**
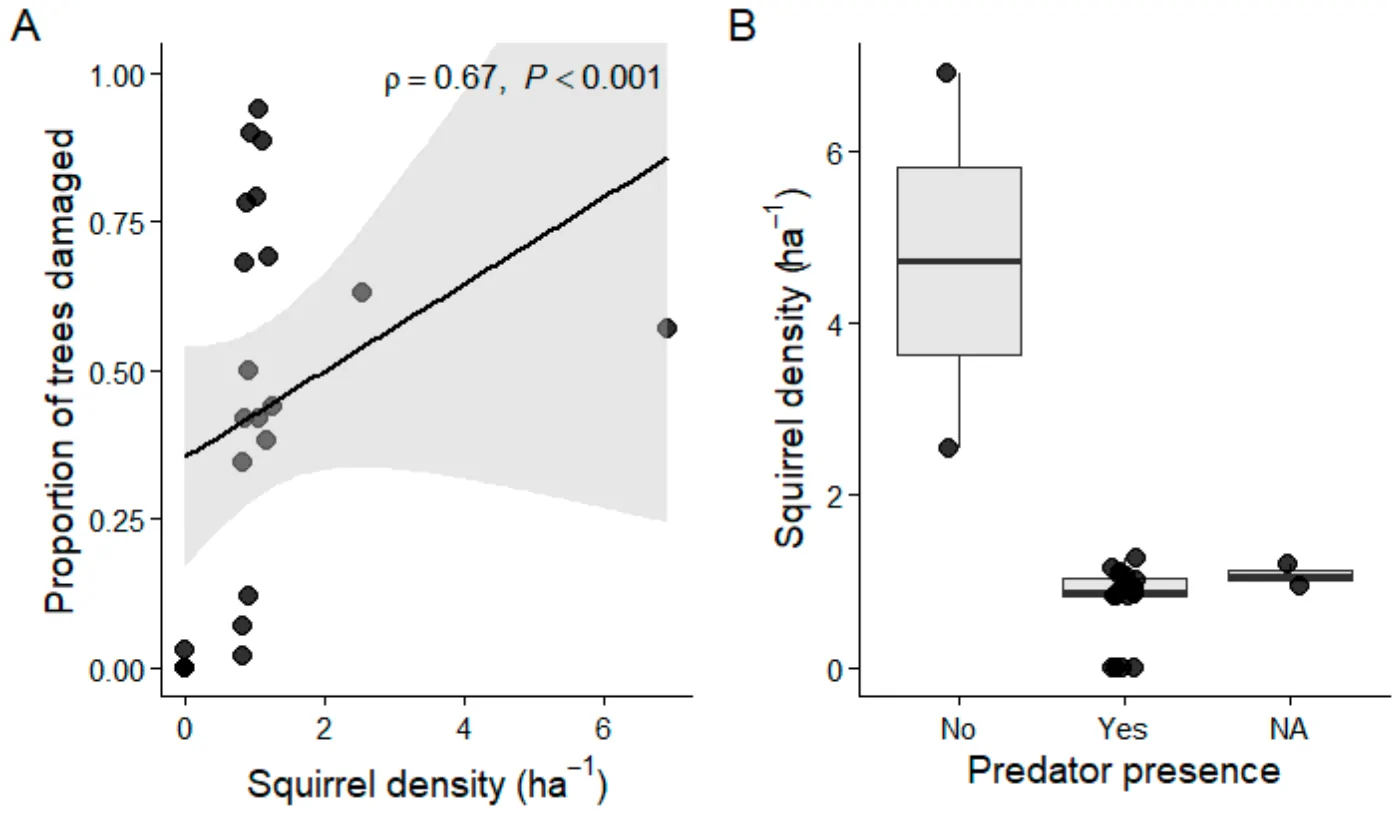
(**A**) Squirrel density (individuals ha^−1^) at sites with and without predator presence (including sites where squirrel density was estimated at 0). Boxplots show the median and interquartile range, whiskers extend to 1.5× the interquartile range, and points represent individual sites. (**B**) Relationship between squirrel density and the proportion of trees damaged. Points represent individual sites, the solid line indicates the fitted linear regression, and the shaded area represents the 95% confidence interval. The inset reports Spearman’s rank correlation coefficient (ρ) and associated *p*-value.

**Table 1:** Categories and description of grey squirrel damage recorded for each tree surveyed.

Information	Category	Description
Damage Age	Fresh	Assumed to have been caused within the last year Wounds show no signs of healing Exposed tissue bright Wound has sharp edges Fresh bark chips may be present on the ground nearby
	Old	Assumed to have been caused more than one year previously Wounds show signs of healing Wounds have rounded or rolled over edges Exposed tissue dark

**Table 2:** The grey squirrel density (individuals/hectare) and record of predator (goshawk and pine marten) presence at all surveyed sites across both years of the study.

Year	Site ID	Density (Individuals/Hectare)	Goshawk Present	Pine Marten Present	Predator Present
2023	2023_A	0.936	Unknown	No	NA
	2023_B	1.051	Yes	No	Yes
	2023_C	6.902	No	No	No
	2023_D	0.909	Yes	Yes	Yes
	2023_E	1.057	Yes	No	Yes
	2023_F	1.194	No	No	No
	2023_G	1.153	Unknown	No	NA
	2023_H	0.909	Yes	No	Yes
	2023_I	2.535	Yes	Yes	Yes
	2023_J	1.113	Yes	No	Yes
	2023_K	1.265	Yes	No	Yes
2024	2024_A	0.824	Yes	Yes	Yes
	2024_B	0.827	Yes	Yes	Yes
	2024_C	0.838	Yes	Yes	Yes
	2024_D	0	Yes	Yes	Yes
	2024_E	0.857	Yes	Yes	Yes
	2024_F	0.889	Yes	Yes	Yes
	2024_G	1.017	Yes	Yes	Yes
	2024_H	0	Yes	Yes	Yes
	2024_I	0	Yes	Yes	Yes
	2024_J	0.821	Yes	Yes	Yes
	2024_K	0	Yes	Yes	Yes

## Data Availability

All data are available on Zenodo: https://zenodo.org/records/21789772?token=eyJhbGciOiJIUzUxMiJ9.eyJpZCI6ImIwZDIzMzA4LTg5YWMtNGVjNS04YmE0LWE2MzRlODQxMDdjNSIsImRhdGEiOnt9LCJyYW5kb20iOiJkMTRlYjA1MTQ2MWJkMzQ2ZTU4ZGEyOWU1MzZjMzlkMyJ9.vB5OJmnUmDbzrJ9Nziuqy6Zmewbhlt6ir5kA-M_ETD55ECNAoWx5RnHtVWCs3RKELMR0iGLoiF7pOpIQ6827sw (accessed on 3 August 2026).

## Supplementary Materials

The following supporting information can be downloaded at: https://www.mdpi.com/article/10.3390/ani16213394/s1, Figure S1: example study site layout; Table S1: summary information for each site used.
