# Supplementary material for: Grey Squirrel Density and Damage in Relation to the Presence of Predators
Source: Animals (Basel). 2026 Oct 27;16(21):3394. doi: 10.3390/ani16213394 (PMC13761971; doi:10.3390/ani16213394)
Supplement: Supplementary file 1 [file animals-16-03394-s001.zip › animals-4512502-supplementary.pdf]

Grey squirrel density and damage in relation to the presence of predators – Supplementary Information

Cally Ham*[1], Eleanor R. Scopes[2,3], Nicola Rae[4], Josh Weaver[1], Robin Gill[1]

1. Forest Research, Alice Holt Lodge, Farnham, UK
2. Forest Research, Forestry Commission National Office, Bristol, UK
3. Environment and Sustainability Institute, University of Exeter, Penryn, UK
4. Forest Research, Northern Research Station, Edinburgh, UK

*Corresponding author: cally.ham@forestresearch.gov.uk

Table S1 Summary information for each site used. Estimated squirrel density uses the methodology published by Beatham et al 2023 [1] and the percentage tree damage uses the Nearest Neighbour method [2]

Site ID	Area (ha)	Predominant Tree Age	Predominant Tree Species	Goshawk Nests Within 5km	Pine Marten Presence	Total Images Recorded
2023_A	15	34	Beech	Unknown	None	1347
2023_B	5	17	Oak	9	None	1154
2023_C	6	25	Oak	0	None	10165
2023_D	3	20	Oak	14	Low	384
2023_E	10	26	Beech	1	None	409
2023_F	10	25	Oak	0	None	8696
2023_G	7	32	Beech	Unknown	None	2814
2023_H	6	23	Beech	1	None	398
2023_I	3	14	Oak	14	Low	701
2023_J	8	23	Sycamore	0	None	5308
2023_K	10	29	Beech	10	None	7695
2023_L	4	37	Oak	5	None	1566
2024_A	12	18	Oak	29	Medium	39442
2024_B	8	20	Oak	15	High	54171
2024_C	7	20	Oak	12	Low	10597
2024_D	9	13	Oak	13	Medium	22776
2024_E	8	17	Oak	26	High	11456
2024_F	10	37	Beech	15	None	21139
2024_G	7	19	Oak	5	Low	12939
2024_H	5	39	Beech	8	Medium	7940
2024_I	10	11	Oak	25	High	25876
2024_J	7	15	Oak	1	Medium	18247
2024_K	10	20	Oak	6	High	13580
2024_L	8	15	Oak	39	Low	14353

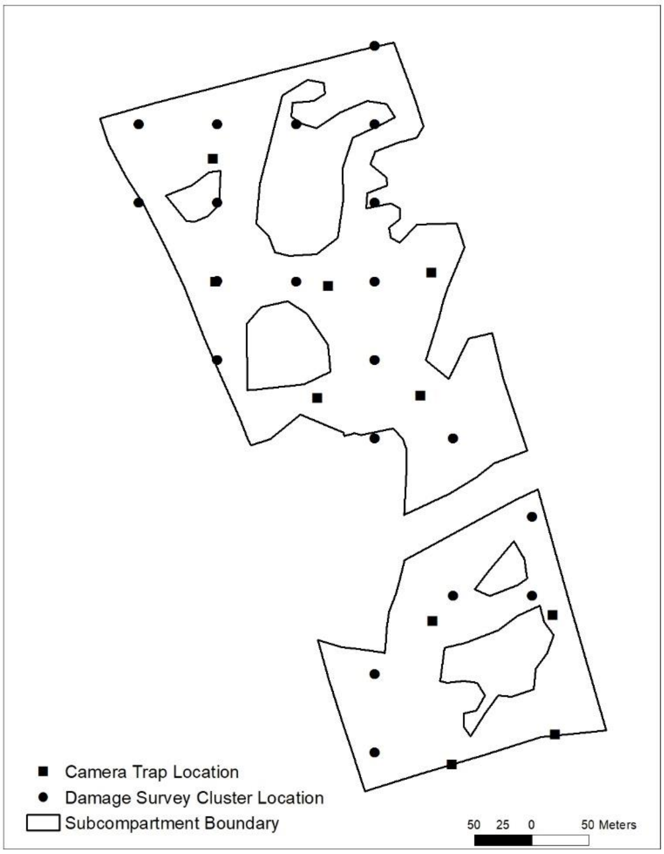

Figure S1 Example survey layout within a study site detailing the locations of summer season camera trapping and winter season tree damage assessments

References

1. Beatham, S.E.; Stephens, P.A.; Coats, J.; Phillips, J.; Massei, G. A Camera Trap Method for Estimating Target Densities of Grey Squirrels to Inform Wildlife Management Applications. *Front. Ecol. Evol.* **2023**, *11*, doi:10.3389/fevo.2023.1096321.

2. Pepper, H. *Nearest Neighbour Method for Quantifying Wildlife Damage to Trees in Woodland*; 1998;
